# Consensus recommendations on therapeutic hypothermia after minimally invasive intracerebral hemorrhage evacuation from the hypothermia for intracerebral hemorrhage (HICH) working group

**DOI:** 10.3389/fneur.2022.859894

**Published:** 2022-08-17

**Authors:** Turner S. Baker, Christopher P. Kellner, Frederick Colbourne, Fred Rincon, Rainer Kollmar, Neeraj Badjatia, Neha Dangayach, J. Mocco, Magdy H. Selim, Patrick Lyden, Kees Polderman, Stephan Mayer

**Affiliations:** ^1^Icahn School of Medicine at Mount Sinai, Sinai BioDesign, New York, NY, United States; ^2^Department of Neurosurgery, Icahn School of Medicine at Mount Sinai, New York, NY, United States; ^3^Department of Psychology, University of Alberta, Edmonton, AB, Canada; ^4^Department of Neurology, Thomas Jefferson University Hospital, Thomas Jefferson University, Philadelphia, PA, United States; ^5^Department of Neurology, University Hospital Erlangen, Friedrich-Alexander-University Erlangen-Nürnberg (FAU), Erlangen, Germany; ^6^Department of Neurology and Neurological Intensive Care, Darmstadt Academic Teaching Hospital, Darmstadt, Germany; ^7^Department of Neurology, University of Maryland School of Medicine, Baltimore, MD, United States; ^8^Department of Neurology, Beth Israel Deaconess Medical Center, Harvard University, Boston, MA, United States; ^9^Department of Physiology and Neuroscience, Keck School of Medicine, Zilkha Neurogenetic Institute, University of Southern California, CA, United States; ^10^United Memorial Medical Center, Houston, TX, United States; ^11^Westchester Medical Center Health Network, Valhalla, NY, United States; ^12^Department of Neurology, New York Medical College, Valhalla, NY, United States; ^13^Department of Neurosurgery, New York Medical College, Valhalla, NY, United States

**Keywords:** therapeutic hypothermia, targeted temperature management, intracerebral hemorrhage, intracranial pressure, perihematomal edema, minimally invasive surgery recommendations for focal hypothermia in ICH

## Abstract

**Background and purpose:**

Therapeutic hypothermia (TH), or targeted temperature management (TTM), is a classic treatment option for reducing inflammation and potentially other destructive processes across a wide range of pathologies, and has been successfully used in numerous disease states. The ability for TH to improve neurological outcomes seems promising for inflammatory injuries but has yet to demonstrate clinical benefit in the intracerebral hemorrhage (ICH) patient population. Minimally invasive ICH evacuation also presents a promising option for ICH treatment with strong preclinical data but has yet to demonstrate functional improvement in large randomized trials. The biochemical mechanisms of action of ICH evacuation and TH appear to be synergistic, and thus combining hematoma evacuation with cooling therapy could provide synergistic benefits. The purpose of this working group was to develop consensus recommendations on optimal clinical trial design and outcomes for the use of therapeutic hypothermia in ICH in conjunction with minimally invasive ICH evacuation.

**Methods:**

An international panel of experts on the intersection of critical-care TH and ICH was convened to analyze available evidence and form a consensus on critical elements of a focal cooling protocol and clinical trial design. Three focused sessions and three full-group meetings were held virtually from December 2020 to February 2021. Each meeting focused on a specific subtopic, allowing for guided, open discussion.

**Results:**

These recommendations detail key elements of a clinical cooling protocol and an outline for the roll-out of clinical trials to test and validate the use of TH in conjunction with hematoma evacuation as well as late-stage protocols to improve the cooling approach. The combined use of systemic normothermia and localized moderate (33.5°C) hypothermia was identified as the most promising treatment strategy.

**Conclusions:**

These recommendations provide a general outline for the use of TH after minimally invasive ICH evacuation. More research is needed to further refine the use and combination of these promising treatment paradigms for this patient population.

## Introduction

### Intracerebral hemorrhage and minimally invasive surgical evacuation

Intracerebral hemorrhage is characterized by bleeding within the parenchyma of the brain. As blood accumulates in the parenchyma, direct mechanical injury damages surrounding neurons and increases intracranial pressure (ICP). This increased pressure leads to reduced cerebral perfusion and additional ischemic injury (1). Hematoma expansion occurs in 33% of patients within the first 24 h, substantially worsening long term functional outcome (2).

Even after the clot has stabilized, additional neurological damage is caused through secondary injury induced by inflammation, neurotoxicity, and other mechanisms. This secondary injury, which manifests visually as perihematomal edema (PHE), comprises both vasogenic and cytotoxic elements and leads to reduced perfusion, disrupted autoregulation (3), and cell death (4, 5). PHE develops rapidly after the bleed, evolving over three phases (3). First, the coagulation cascade is activated, resulting in elevated interstitial osmotic pressure, causing fluid from the surrounding vasculature to flow into the parenchyma, resulting in an additional mass effect. Throughout the second phase, the elevated presence of thrombin causes PHE development and expansion through a diverse set of mechanisms, including increased inflammation via the complement cascade and increased permeability of the blood-brain barrier (6, 7). As the clot continues to break down, hemoglobin accumulates, leading to inhibition of adenosine triphosphate activity, increased hydroxyl radicals, and neuronal death (8). PHE volume has been shown to peak between 1 and 2 weeks after hemorrhage onset in patients (9, 10).

Recent studies have suggested a potential benefit for minimally invasive surgical (MIS) evacuation, a procedure in which a small craniotomy or burr-hole is made and a device is stereo tactically guided into the blood clot to permit evacuation through a small channel under enhanced visualization (11, 12). The benefits of MIS-ICH evacuation function primarily through the reduction of mass effect and the mitigation of the secondary effects of hematoma toxicity on the surrounding brain. The recently published Minimally Invasive Surgery plus rt-PA for Intracerebral Hemorrhage III trial (MISTIE III) failed to demonstrate a significant benefit of minimally invasive catheter drainage with thrombolysis over the standard-of-care in its primary endpoint of 6-month modified Rankin Scale (mRS) (13). Secondary endpoints, however, suggested that catheter drainage with thrombolysis may decrease mortality overall and may improve functional outcome for patients in whom the vast majority of the clot is removed by the end of treatment. Minimally invasive evacuation has been linked to a decrease in edema, suggesting a potential neuroprotective mechanism of action for hematoma removal (10, 13, 14).

### Therapeutic hypothermia and targeted temperature management

Hypothermia is one of the earliest and most thoroughly studied neuroprotective strategies, exerting its effects through multiple mechanisms (15–17). Acutely, hypothermia reduces metabolic rate and the release of excitatory neurotransmitters, and decreases glucose metabolism (18). Subacutely, it reduces cell-mediated inflammation with a reduction in inflammatory markers (TNF-a, IL-1b, MMP-2, and MMP-9), down-regulation of pro-apoptotic BAX gene expression, and up-regulation of anti-apoptotic BCL-2 expression (19–21).

Systemic TH is the practice of inducing hypothermia through the reduction of body-core temperature, thereby achieving hypothermia of all organs including the target organ, the brain. Systemic hypothermia can be achieved by multiple modalities including surface cooling (e.g., Arctic Sun, Criticool, Flexipad, IQool), endovascular cooling via venous catheters (e.g., Quattro, Cool Line, ICY). No substantial evidence has demonstrated efficacy or superiority of any modality (22, 23).

The application of systemic TH has been studied in multiple conditions that result in neurological injury including post-cardiac arrest (24), traumatic brain injury (25, 26), subarachnoid hemorrhage (27), cerebral edema from hepatic encephalopathy (28), and ischemic stroke (29). The most recent large RCT assessing the use of TH following cardiac arrest saw no improvement between systemic TH and standard care, upending a longstanding debate over the optimal target temperature for patients post-cardiac arrest (30, 31). While this newest finding may diminish excitement in the field of critical care, TH retains promising potential. Studies have also demonstrated the importance of a controlled rewarming period following systemic TH, with an increase of 0.25°C/hour as a common rate (30, 32).

Systemic TH carries specific risks related to the lack of organ-specific targeting. Shivering is common amongst systemically cooled patients, thereby requiring the use of high-dose sedatives to minimize delays in reaching target temperatures. Increased rates of infection, particularly pneumonia, are significantly associated with systemic TH, which may be partially explained by TH impairing leukocyte function and T-cell activity (33–37). Other side effects include bradyarrhythmias, coagulopathies, and metabolic impairments (38, 39).

Given the limitations of systemic TH, the development of regional cooling devices and protocols have sought to avoid the side effects of full-body cooling while still incurring the benefits of TH. Surface cooling mechanisms including cooling caps and neckbands have been found to successfully achieve parenchymal temperatures, measured with tympanic probes, as low as 33°C while maintaining core normothermia (40). Intranasal cooling, where circulated air or spray induces rapid evaporation and subsequent cooling within the nasal cavity, has been proven feasible and safe in stroke patients (41–44). Intracarotid infusion of cold saline also appears to effectively induce targeted cerebral TH while reducing infarction size following ischemic stroke (45).

#### Therapeutic hypothermia for ICH

Multiple meta-analyses of preclinical studies have shown the ability of TH to reduce PHE and improve neurobehavioural scores, however, there has yet to be a definitive human study demonstrating efficacy in a clinical setting (46, 47). Several historically controlled studies did find significant PHE reduction in response to systemic cooling but were also associated with increased rates of complications, such as pneumonia, and ultimately found no improvement in functional outcomes (48, 49). The results of the first US-based RCT looking at TH in ICH, the TTM-ICH trial (50), will be released soon and the only other published protocol of TH in ICH, the CINCH trial (51), is still recruiting patients, but treatment is no longer randomized. Neither of these studies will likely hit their recruitment target of 50 patients, meaning that further studies investigating the use of TH in ICH should be designed and conducted.

ICH would benefit from the neuroprotection induced through TH, however, given the fragility of the patient population, it is imperative that particular considerations be taken to optimize safety and efficacy. In the case of cardiac arrest and ischemic stroke, treatment is deployed immediately, as patients are frequently in near-catatonic states and substantial evidence indicates the benefits of rapid treatment. The deployment of TH is easily built into these high-level emergencies, as multiple physicians are continuously involved with therapy. Compared to patients presenting with ischemic stroke, ICH patients are considered a lower level of emergency due to the lack of effective treatment, resulting in treatment paradigms focusing on stabilization and monitoring. The induction of early systemic hypothermia in ICH inherently elevates the level of emergency in these patients, increasing the likelihood of complications and requiring additional expert monitoring. TH-induced coagulopathy is also a particular concern, as demonstrated in animal ICH studies (52).

The use of focal cooling may avoid the negative side-effects of systemic cooling, yet the ability to cool deep within the brain via surface or intranasal cooling methods is currently in question. The skull is an efficient insulator, hampering the ability for surface cooling to effectively cool areas of deep injury. High rates of cerebral blood perfusion act to introduce heat to focal cooling systems, potentially limiting the ability of surface or nasal cooling to penetrate to deeper bleeds or areas of injury.

The development of MIS techniques to treat ICH introduces a unique avenue to induce focal cooling, as accessing the bleed to perform evacuations inherently allows for access to the primary site of injury. The induction of intracavitary cooling, cooling initiated from within the cavity created during clot evacuation, would efficiently target the PHE core, but may also incur additional risks due to its intracranial deployment. This combination therapy addresses the major destructive mechanisms of ICH by reducing mass effect, removing the iron-rich clot, and reducing the local neuroinflammatory cascade. Multiple clinical trials have already been conducted in China, where MIS-ICH evacuation has already been widely adopted, indicating a benefit of postoperative cooling compared to control (53).

Herein, we present emerging recommendations from the Therapeutic Hypothermia for ICH (‘HICH') Working group. This includes a recommended cooling protocol to demonstrate and optimize TH in ICH that has been evacuated with minimally invasive techniques. Specific procedures for cooling initiation and maintenance are described, as are optimal trial elements.

## Methods

An international panel of experts on TH and ICH was convened to form a consensus on critical elements of a focal cooling protocol and the outline of a systematic pathway to develop TH as a potential adjunctive therapy in ICH patients undergoing MIS evacuation. The full group met virtually for three sessions from December 2020 to February 2021, with three additional meetings with session leaders prior to each full meeting. Each meeting focused on a specific topic and was led by group members that specialized in the focus. Prior to each meeting members were asked to complete a questionnaire outlining their views, which were combined into a short presentation to guide discussion. Meeting topics included: (1) General outlook of the field [TB, CK], (2) best cooling procedures for focal use of TH in conjunction with ICH evacuation [FC, KP], and (3) key trial design characteristics for assessing benefit through feasibility and pivotal trials [RC, RK]. Meetings were open-forum, allowing for open discussion on each assigned topic. All discussions were recorded and an outline of each discussion was written and shared with the entire group for review.

## Recommended cooling procedure

**Figure 1:**
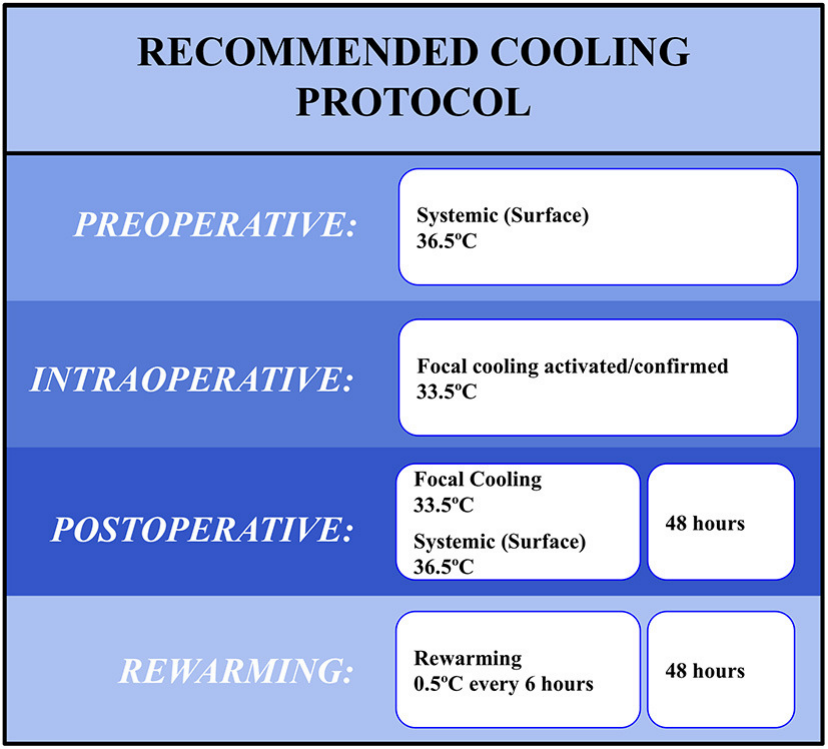
HICH recommended cooling protocol for use with ICH evacuation.

The following recommendations (Figure 1) are based upon current clinical knowledge, which is lacking in pivotal randomized controlled trials. Many of these recommendations, therefore, prioritize safety over efficacy, resulting conservative recommendations.

### Systemic and local cooling strategies

Historically, clinical use of TH with ICH has involved systemic hypothermia despite the inherent limitations of secondary complications. The recommended cooling protocol implements a combination of both systemic nor-mothermia with localized intracerebral hypothermia. This technique has already gained traction in ischemic stroke, where endoscopic localized cold infusions are used to improve reperfusion injury. Numerous preclinical ICH studies have also connected localized cooling to significant reduction in edema. This should allow for the induction of inflammatory inhibition while minimizing the negative effects associated with non-specific organ cooling. Further innovation is required as there is currently no approved clinical device or mechanism to induce local cooling following ICH.

### Preoperative cooling

We recommend that mild systemic cooling be initiated while the patient is being prepared for surgery, immediately before the initiation of anesthesia. The neuro-inflammatory pathway associated with ICH initiates immediately upon injury, and early targeted temperature management to stem the initial cascade prior to evacuation may be beneficial. While some preclinical studies have demonstrated increased hematoma growth in response to profound cooling, meta-analyses have routinely failed to show a significant relationship between cooling and hematoma expansion, particularly when cooling is kept above 33°C (52, 54). It makes sense to utilize the surgery preparation stage as a de-risked initiation period for the cooling, where ICH confirmation, physician presence, and hematoma stability will be confirmed prior to cooling initiation.

We recommend the patient be cooled systemically with a controlled feedback loop to nor-mothermic levels with a target temperature of 36.5°C. Surface cooling appears the optimal mechanism as normothermia in most cases does not induce shivering, rapid cooling is not required, and therefore reduces the need for endovascular cooling mechanisms. Temperature selection is intended to (1) reduce fever burden, (2) minimize the risk of temperature-induced coagulopathy, and (3) maximize the cooling ability of the localized hypothermia to be induced intraoperatively.

### Intraoperative cooling

Systemic normothermia should be continued throughout the surgical procedure via the same cooling techniques employed preoperatively. Localized intracerebral cooling should be initiated immediately following completion of the clot evacuation but prior to the removal of the endoscope. This will allow for direct monitoring of the hematoma cavity for TH-induced re-bleeds and confirmation of successful cooling of the injury core. Local cooling and systemic normothermia should continue until the procedure is completed and through postoperative care.

We recommend a target local temperature of 33.5°C. Cooling should be induced rapidly, but with a lower boundary of 33°C as preclinical data suggests that coagulopathy issues occur at that temperature (55). The continued systemic normothermia will assist any mechanism used to induce localized cooling by reducing the potential perfusion of <36.5°C blood through the brain.

### Post-operative cooling

Both intracerebral cooling and systemic normothermia should be continued at target temperatures of 35 and 36.5°C, respectively. Cooling should be maintained for 48 h, which fits within common neurological recovery periods following MIS-IHC evacuation. Upon the completion of the cooling period, rewarming should increase intracranial temperature at a rate of 0.5°C every 6 h. This rewarming rate should be done with special attention paid to intracranial pressure. The entire protocol should ideally conclude 4 days after hematoma evacuation.

## Recommended clinical development program patient criteria and outcome measures

ICH is a complex condition and the best treatment strategies for evacuation are still under investigation. It is therefore crucial that trials assessing the use of TH in combination with hematoma evacuation be well-considered and thorough. Here we outline a recommended protocol for clinical development program testing the impact of the previously described cooling protocol, as well as a series of future clinical trials to iteratively demonstrate the clinical effects of TH for ICH.

### Recommended inclusion/exclusion criteria

**Figure 2:**
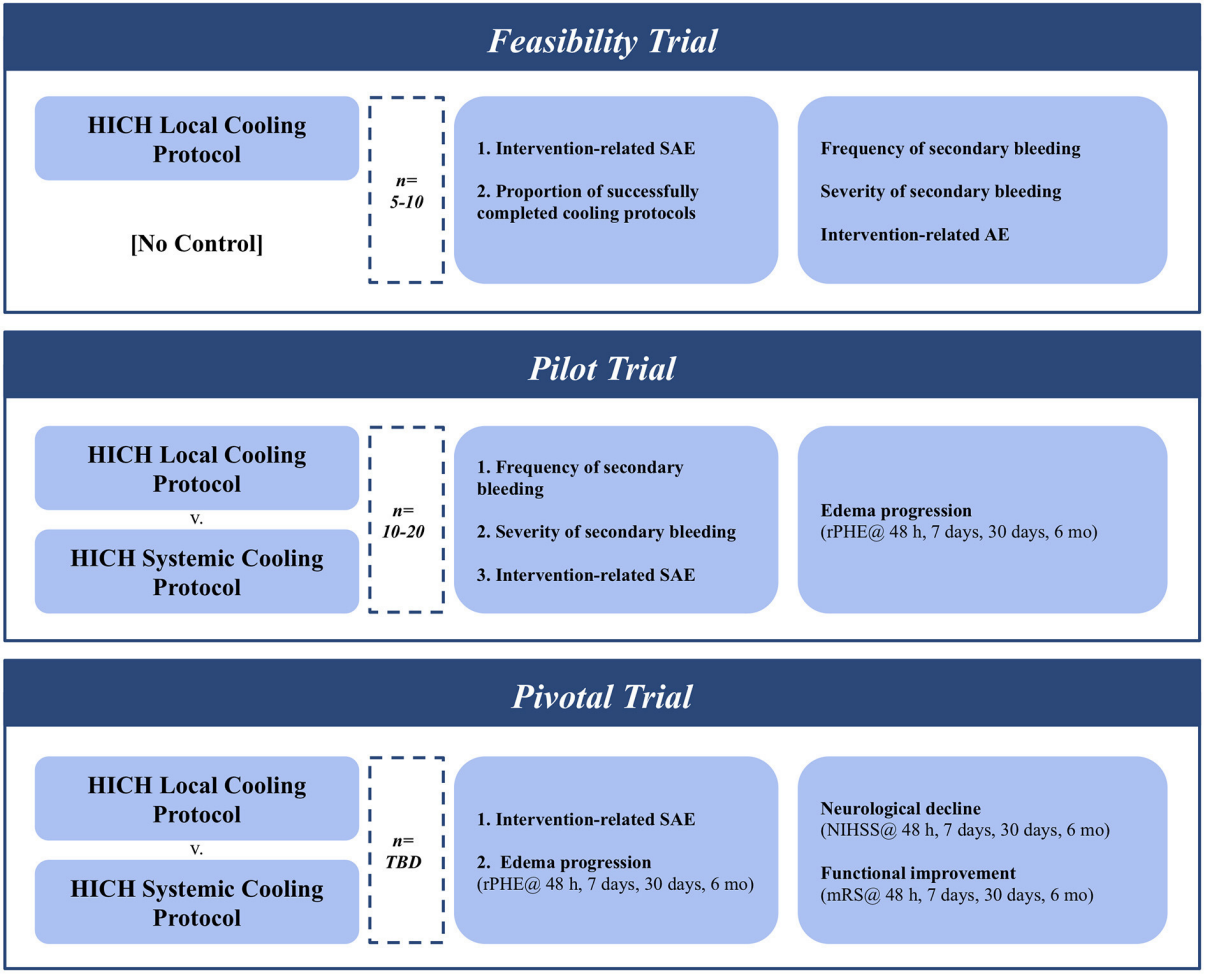
Iterative clinical trial design for testing & establishing optimal cooling protocols for ICH with MIS evacuation.

The recommended inclusion and exclusion criteria are intentionally heterogeneous to allow for a wider variety of clinical scenarios that may benefit most from this treatment paradigm. This will also ameliorate recruitment struggles, which has led to extended periods for previously published ICH cooling trials (50, 51). Recruitment ability may be further improved by the combination therapy with hematoma evacuation, which has been successful in recruiting ample sample sizes across a variety of studies (56–59) (Figure 2).

Patients with spontaneous supratentorial ICH under age 80 who meet all criteria should be included in the study. This matches standard eligibility criteria for ICH evacuation at established institutions, where most have a cap of 80 years which also has been used as an inclusion criterion in most previous studies. There is no clinical evidence that older individuals suffer more negative effects of systemic or focal TH, and with a majority of ICH patients being over the age of 60 it will be crucial to include older individuals to achieve successful sample sizes.

Hematoma size quantification must be conducted prior to patient enrollment, with a recommended minimum bleed size of 20 mL required for trial inclusion. This is a common threshold in standard evacuation protocols, as well as prior systemic TH for ICH trials without evacuation (51).

A minimum Glasgow Coma Scale score of 6 or more is recommended. This is more inclusive than past TH trial protocols but represents the prevailing wisdom within ICH evacuations given the acute nature of treatment. Standard procedures at institutes frequently performing evacuations require a GCS score of 4 or more. It is possible that active profound cooling of certain brain regions may negatively affect immediate postoperative neurological scores, therefore confounding results, so an upper limit of GCS may be required for future trials.

While the value of early evacuation is still under investigation, it is clear that TH appears most effective when introduced early after neurologic injury. As certain institutes performing regular evacuations are able to achieve insult-to-evacuation times below 12 h, it is recommended that the initiation of TH for early feasibility and pilot trials be set to 12 h. It is unclear if all institutions would be able to achieve this time course for a larger, multisite trial, so later studies may extend initiation delay to a 24-h maximum. There is preclinical evidence of increased bleeding in response to early induction of low (<33°C) temperatures following ICH induction, however, hematoma evacuation allows for direct control of potential temperature-induced secondary bleeding. The recommended target temperature is also higher than prior research showing increased frequency or severity of re-bleeds.

### Experimental arms through trial rollout

Despite extensive preclinical data there has yet to be a controlled study assessing the use of focal hypothermia to improve ICH outcomes in patients (60, 61). An initial trial needs to be conducted to determine the effect of focal cooling in conjunction with ICH evacuation. This single-arm early feasibility study (*n* = 5–10) should focus primarily on the functionality of the cooling mechanism by quantifying the amount of intracranial cooling achieved. Multiple modalities exist to measure this effect, including implantable temperature probes and real-time magnetic resonance thermometry. This early trial should also track device-related severe adverse events (SAE). Cooling-induced coagulopathy leading to secondary bleeding or hematoma instability is a key concern with focal cooling after evacuation, and symptomatic and asymptomatic re-bleeds should be closely tracked. Recruitment rates will serve to validate the previously outlined inclusion/exclusion criteria.

Having demonstrated the feasibility of local hypothermia in the setting of post-ICH evacuation, a larger pilot trial (*N* = 10–20) will be required to establish safety and efficacy parameters of the proposed protocol. The frequency and severity of secondary bleeding should again be tracked closely and reported on by the study team, as should any other anticipated adverse events. Secondary outcomes should closely track the expansion of PHE volume relative to the hematoma size (rPHE). This should be tracked at the end of active cooling (48 h), at discharge from the neurosurgical ICU (7 days), as well as at a 7 and 30-day follow-up.

## Discussion

Closely controlled hypothermia remains a highly promising avenue for improving outcomes after ICH. The mechanisms behind hematoma removal and brain cooling should theoretically work additively or synergistically, and it is crucial that the field form a consensus on optimal cooling protocols for ICH patients with the data generated from past studies. It is important to note that the recommended cooling protocol described here is designed to minimize risk, and the results of future studies will likely augment and improve both safety and efficacy outcomes. Two primary parameters have been identified in most need of additional research: target temperature and duration of cooling. The combination of these parameters describes a TH “dose.” The use of TH as a treatment paradigm is unique in that a device-centric, physical therapeutic must be dosed appropriately for each pathology. Systemic TH dosage for post-cardiac arrest patients is still under debate, and it is likely that the use of TH in postoperative ICH patients must navigate a similar period of trials, analysis, and debate.

Local cooling in conjunction with evacuation has been identified by this working group due to the unique access of ICH evacuation and the synergistic nature of the mechanisms; however, there is no currently available technology to safely and effectively induce this intervention, suggesting the need for further innovation. The local target temperature of 33.5°C was selected to minimize intracavitary coagulopathy risk. The use of highly localized intracranial cooling could allow for more profound cooling as colder temperatures induce significant vasoconstriction, which may offset any temperature-induced coagulopathy (62). Temperature-induced coagulopathy has been shown to be directly correlated to specific temperatures, however significant changes in bleeding have previously been found to be as low as 24°C, suggesting it may be beneficial to induce intracranial TH at a target temperature in the upper twenties (55). Alternatively, deep cooling well-below temperatures that affect coagulopathy may result in a higher frequency of adverse events, namely post-evacuation rebleeds. Preclinical data have found moderate (<35°C) cooling to result in hematoma expansion, however, those studies often induced cooling during active bleeding or within an hour since the bleed and did not pair cooling therapies with any evacuation protocols (63). Stepwise or dose-finding studies to find an optimal balance between safety and efficacy as a function of temperature are needed.

A cooling duration of 48 h was recommended as it spans the length of key PHE periods, which have been found to expand most rapidly within 6 h of hematoma formation, peaking at approximately 48 h post incidence. Current literature does not conclusively indicate the effect early cooling will have on PHE expansion, however rapid, short-term cooling has been shown to inhibit the development of inflammatory cascades across a variety of pathologies. Future research may show that early induction of TH does enough to stem the evolution of the PHE and associated inflammatory pathways, and therefore would reduce required cooling duration; this would match similar findings with ischemia when treatment occurs during or immediately following ischemia compared to delayed cooling.

An injury-to-cooling threshold of 12 h was recommended in an attempt to maximize the overall effect of the cooling on initial inflammation reduction. Ultimately this time point will be decided by the ability of facilities to engage in rapid diagnosis and patient transfer as well as the results of ongoing clinical studies evaluating the safety, feasibility, and efficacy of early evacuation (12, 64). Extensive delays between the initial bleed and the subsequent evacuation and cooling may prove to substantially weaken the benefits of TH. Some preclinical data have suggested that prolonging the cooling period has been found to make up for increased delays to cooling in ischemic stroke cases, however, that data has not been confirmed in a single study assessing the relationship between prolonged cooling and intervention delay (65). It will be key for cooling protocols to not negatively affect standard process of care metrics, such as first mobilization, mechanical ventilator time, sedation level, and total time within the ICU. Prolonged or profound cooling may delay these short-term metrics, and all trials assessing the use of cooling should tightly control these elements to ensure accurate analysis.

## Data availability statement

The original contributions presented in the study are included in the article/supplementary material, further inquiries can be directed to the corresponding author/s.

## Author contributions

TB, CK, and SM organized and hosted meetings. All authors participated in discussions and as well as drafting and editing of the manuscript.

## Conflict of interest

Author NB has received research funding from Becton Dickenson, Maryland Industrial Partnerships, Department of Defense. Author TB owns equity in a start-up investigating mechanisms of local cooling for ICH. Authors TB and CK are listed inventors in IP relating to cooling after ICH. The remaining authors declare that the research was conducted in the absence of any commercial or financial relationships that could be construed as a potential conflict of interest. The reviewer BE declared a shared affiliation with the author PL to the handling editor at the time of review.

## Publisher's note

All claims expressed in this article are solely those of the authors and do not necessarily represent those of their affiliated organizations, or those of the publisher, the editors and the reviewers. Any product that may be evaluated in this article, or claim that may be made by its manufacturer, is not guaranteed or endorsed by the publisher.
